# Supplementary material for: How well do you know your mutation? Complex effects of genetic background on expressivity, complementation, and ordering of allelic effects
Source: PLoS Genet. 2017 Nov 22;13(11):e1007075. doi: 10.1371/journal.pgen.1007075 (PMC5718557; doi:10.1371/journal.pgen.1007075)
Supplement: S1 Table — (DOCX) [file pgen.1007075.s001.docx]

**Supplementary Table 1.**

| DNA \ Assay | orew | ore_13 | O_2a33 | O_58d | O_E3 | O_ETX4 | O_G0309 | O_sd1 | O_vg1 | samw | sam_13 | S_2a33 | S_58d | S_E3 | S_ETX4 | S_G0309 | S_sd1 | S_vg1 |
| --- | --- | --- | --- | --- | --- | --- | --- | --- | --- | --- | --- | --- | --- | --- | --- | --- | --- | --- |
| 2102481059 | ? | ? | ? | ? | ? | ? | ? | ? | ? | T:T | T:T | T:T | ? | T:T | T:T | T:T | T:T | T:T |
| 2220229229 | ? | ? | ? | ? | ? | ? | ? | ? | ? | G:G | G:G | G:G | G:G | G:G | G:G | G:G | G:G | G:G |
| 2210699161 | A:A | A:A | A:A | A:A | A:A | A:A | A:A | A:A | C:C | A:A | A:A | A:A | A:A | A:A | A:A | A:A | A:A | A:A |
| 1003024740 | ? | G:G | G:G | ? | G:G | G:G | G:G | G:G | G:G | ? | ? | ? | ? | ? | ? | ? | ? | G:G |
| 1004109484 | ? | C:C | C:C | T:T | C:C | C:C | C:C | C:C | C:C | T:T | T:T | T:T | T:T | T:T | T:T | T:T | T:T | ? |
| 1004435848 | G:G | G:G | G:G | A:A | G:G | G:G | G:G | G:G | G:G | G:G | G:G | G:G | G:G | G:G | G:G | G:G | G:G | G:G |
| 1005197972 | G:G | G:G | G:G | T:T | G:G | G:G | G:G | G:G | G:G | T:T | T:T | T:T | T:T | T:T | T:T | T:T | T:T | T:T |
| 1006199670 | T:T | T:T | T:T | C:C | T:T | T:T | T:T | T:T | T:T | T:T | T:T | T:T | T:T | T:T | T:T | T:T | T:T | T:T |
| 1008051543 | C:C | C:C | C:C | C:C | C:C | C:C | C:C | C:C | C:C | ? | ? | ? | ? | ? | ? | ? | ? | ? |
| 1016468853 | C:C | C:C | C:C | C:C | C:C | C:C | C:C | C:C | C:C | C:C | C:C | C:C | C:C | C:C | C:C | C:C | A:A | C:C |
| 1019513630 | G:G | G:G | G:G | G:G | G:G | G:G | G:G | G:G | G:G | G:G | G:G | G:G | G:G | G:G | G:G | G:G | A:A | G:G |
| 2100935053 | T:T | T:T | T:T | T:T | T:T | T:T | T:T | T:T | T:T | G:G | G:G | G:G | T:T | G:G | G:G | G:G | G:G | ? |
| 2103475351 | ? | ? | ? | ? | ? | ? | ? | ? | ? | T:T | T:T | T:T | ? | T:T | T:T | T:T | T:T | T:T |
| 2103749654 | C:C | C:C | C:C | C:C | C:C | C:C | C:C | C:C | C:C | A:A | A:A | A:A | C:C | A:A | A:A | A:A | A:A | ? |
| 2105147351 | A:A | A:A | A:A | A:A | A:A | ? | A:A | A:A | ? | G:G | G:G | G:G | A:A | G:G | G:G | G:G | G:G | A:A |
| 2105894785 | A:A | A:A | A:A | A:A | A:A | A:A | A:A | A:A | A:A | G:G | G:G | G:G | A:A | G:G | G:G | G:G | G:G | A:A |
| 2106404739 | G:G | G:G | G:G | G:G | G:G | G:G | G:G | G:G | G:G | T:T | T:T | T:T | G:G | T:T | T:T | T:T | T:T | G:G |
| 2107266022 | A:A | A:A | A:A | A:A | A:A | A:A | A:A | A:A | A:A | C:C | C:C | C:C | A:A | C:C | C:C | C:C | C:C | A:A |
| 2109665429 | T:T | T:T | T:T | T:T | T:T | T:T | T:T | T:T | T:T | G:G | G:G | G:G | T:T | G:G | G:G | G:G | G:G | T:T |
| 2110053105 | ? | ? | ? | ? | ? | ? | ? | ? | ? | A:A | A:A | A:A | ? | A:A | A:A | A:A | A:A | ? |
| 2111307108 | ? | ? | ? | ? | ? | ? | ? | ? | ? | T:T | T:T | T:T | ? | T:T | T:T | T:T | T:T | ? |
| 2111348992 | ? | ? | G:G | G:G | G:G | G:G | G:G | ? | G:G | A:A | A:A | A:A | G:G | A:A | A:A | A:A | A:A | G:G |
| 2111392798 | A:A | A:A | A:A | A:A | A:A | A:A | A:A | A:A | A:A | C:C | C:C | C:C | A:A | C:C | C:C | C:C | C:C | A:A |
| 2111404367 | G:G | G:G | G:G | G:G | G:G | G:G | G:G | G:G | G:G | T:T | T:T | T:T | G:G | T:T | T:T | T:T | T:T | G:G |
| 2111635586 | ? | ? | ? | ? | ? | ? | ? | ? | ? | A:A | A:A | A:A | ? | A:A | A:A | A:A | A:A | ? |
| 2111923508 | G:G | G:G | G:G | G:G | G:G | G:G | G:G | G:G | G:G | T:T | T:T | T:T | G:G | T:T | T:T | T:T | T:T | G:G |
| 2111937433 | C:C | C:C | C:C | C:C | C:C | C:C | C:C | C:C | C:C | A:A | A:A | A:A | C:C | A:A | A:A | A:A | A:A | C:C |
| 2112116496 | G:G | G:G | G:G | G:G | G:G | G:G | G:G | G:G | G:G | A:A | A:A | A:A | ? | A:A | A:A | A:A | A:A | G:G |
| 2112303402 | ? | ? | ? | ? | ? | ? | ? | ? | ? | G:G | G:G | G:G | G:G | G:G | G:G | G:G | G:G | ? |
| 2112406358 | C:C | C:C | C:C | C:C | C:C | C:C | ? | C:C | C:C | T:T | T:T | T:T | T:T | T:T | T:T | T:T | T:T | C:C |
| 2112637667 | T:T | T:T | T:T | T:T | T:T | T:T | T:T | T:T | T:T | C:C | C:C | C:C | T:T | C:C | C:C | C:C | C:C | T:T |
| 2114680685 | ? | ? | ? | ? | ? | ? | ? | ? | A:A | G:G | G:G | G:G | G:G | G:G | G:G | G:G | G:G | ? |
| 2117878931 | G:G | G:G | G:G | G:G | G:G | G:G | G:G | G:G | G:G | ? | ? | ? | G:G | ? | ? | ? | ? | G:G |
| 2117895246 | T:T | T:T | T:T | T:T | T:T | T:T | T:T | T:T | T:C | T:T | T:T | T:T | T:T | T:T | T:T | T:T | T:T | T:C |
| 2119110381 | A:A | A:A | A:A | A:A | A:A | A:A | A:A | A:A | A:A | T:T | T:T | T:T | T:A | T:T | T:T | T:T | T:T | T:A |
| 2119216825 | T:T | T:T | T:T | T:T | T:T | T:T | T:T | T:T | ? | T:T | T:T | T:T | T:T | T:T | T:T | T:T | ? | T:A |
| 2119487200 | C:C | C:C | C:C | C:C | C:C | C:C | C:C | C:C | C:C | A:A | A:A | A:A | C:A | A:A | A:A | A:A | A:A | C:A |
| 2202214593 | T:T | T:T | T:T | T:T | T:T | T:T | T:T | T:T | T:T | A:A | A:A | A:A | T:A | A:A | A:A | ? | A:A | T:A |
| 2203732013 | T:T | T:T | T:T | T:T | T:T | T:T | T:T | T:T | T:T | A:A | A:A | A:A | T:T | A:A | A:A | A:A | A:A | ? |
| 2208396532 | C:C | C:C | C:C | C:C | C:C | C:C | C:C | C:C | C:C | G:C | G:C | G:C | C:C | G:C | G:C | G:C | G:C | C:C |
| 2209101838 | C:C | C:C | T:T | C:C | C:C | C:C | C:C | C:C | T:T | C:C | C:C | C:C | C:C | C:C | C:C | C:C | C:C | T:T |
