# Supplementary figures and images for: How well do you know your mutation? Complex effects of genetic background on expressivity, complementation, and ordering of allelic effects
Source: PLoS Genet. 2017 Nov 22;13(11):e1007075. doi: 10.1371/journal.pgen.1007075 (PMC5718557; doi:10.1371/journal.pgen.1007075)

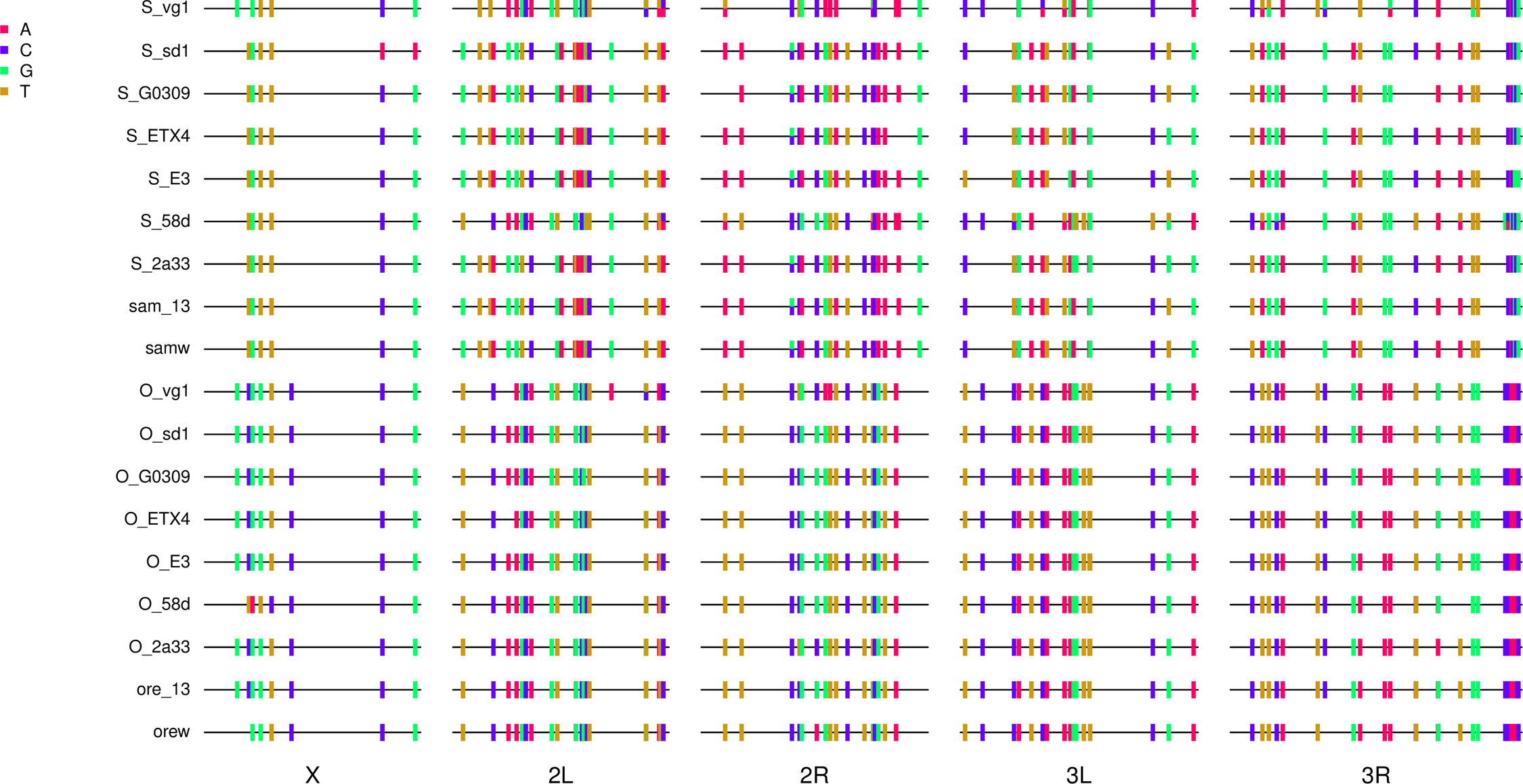

Supplement: S1 Fig — With a few exceptions, introgressions appeared mostly complete, except close to the focal allele. (TIF) [file pgen.1007075.s002.tif]

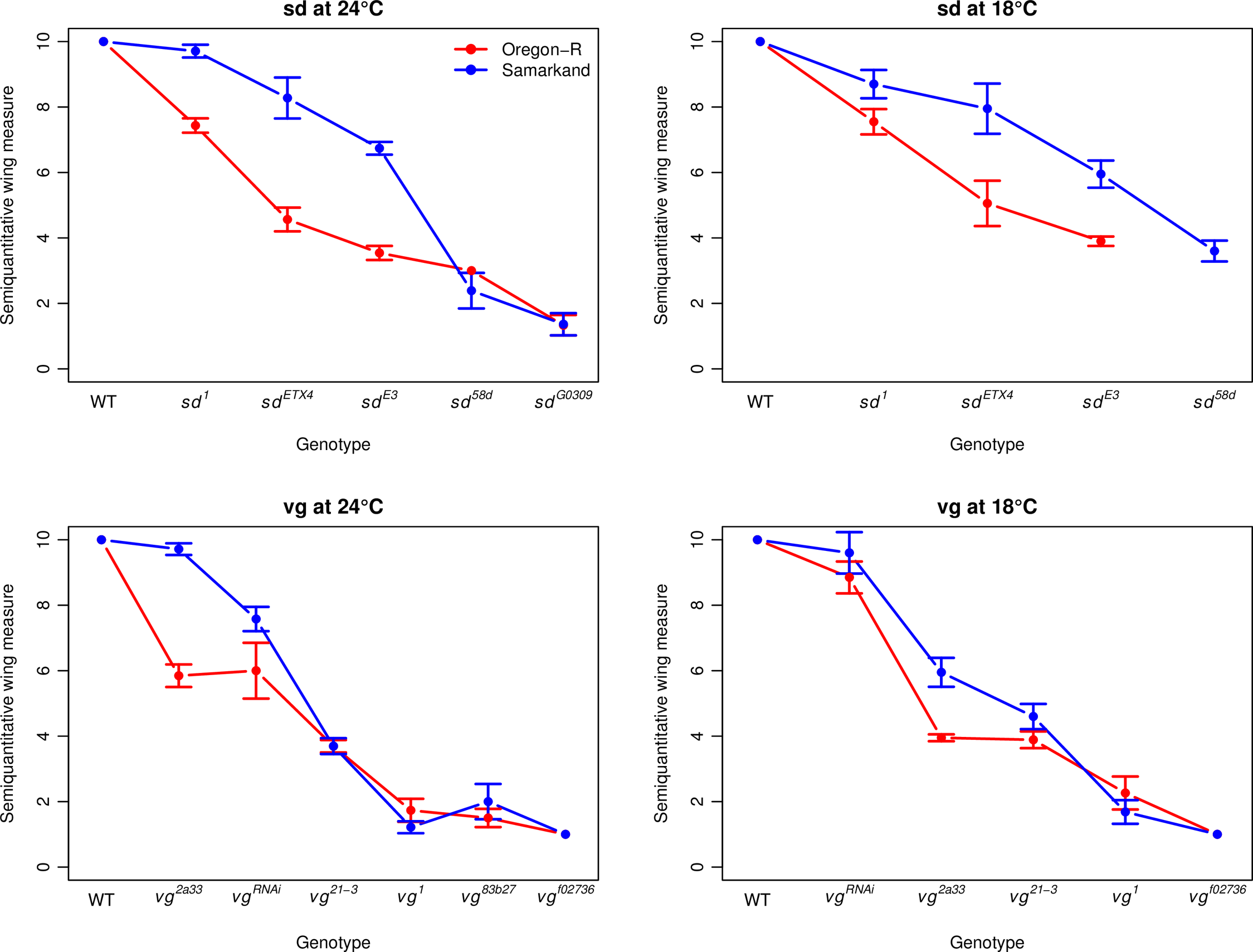

Supplement: S2 Fig — The influences of genetic background and rearing temperature on the expressivity of allelic series in the sd and vg genes, using an ordinal scale as a measure of overall wing phenotype. "Stronger" mutations result in smaller wings. The vgRNAi represents a cross of the UAS-vg.RNAi to NP6333-GAL4 (both alleles introgressed into both genetic backgrounds). Error bars represent 95% confidence intervals. (TIF) [file pgen.1007075.s003.tif]

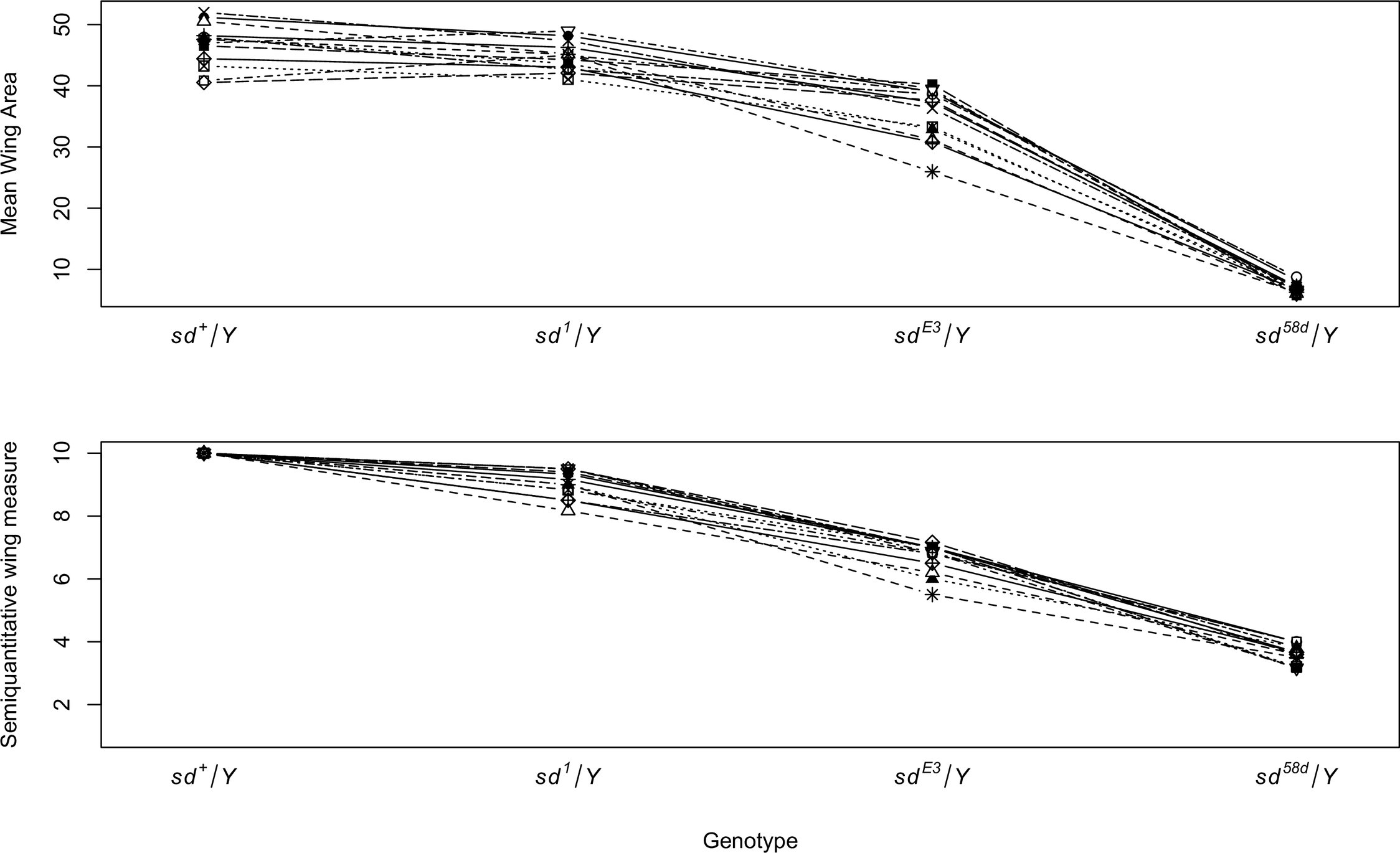

Supplement: S3 Fig — Each line represents the male F1 offspring from crosses from males of one of 16 DGRP strains to females of SAM sd1, SAM sdE3, SAM sd58d (and corresponding SAM wild type). Top panel is the measure of wing area, while the bottom panel uses the semi-quantitative measure of wing morphology. It is worth noting that the experimental design was by necessity different from the primary experiment (where alleles were introgressed and tested in homozygous genetic backgrounds). In this experiment, we are examining the effects of the sd alleles in hemizygous males, for the 16 genetic backgrounds heterozygous over the SAM genetic background. As such the among genetic background variance is far less (as recessive effects will generally not be expressed). Furthermore, we used the SAM background, which showed a generally weaker degree of phenotypic expressivity of mutations, which resulted in less severe phenotypic effects. (TIF) [file pgen.1007075.s004.tif]

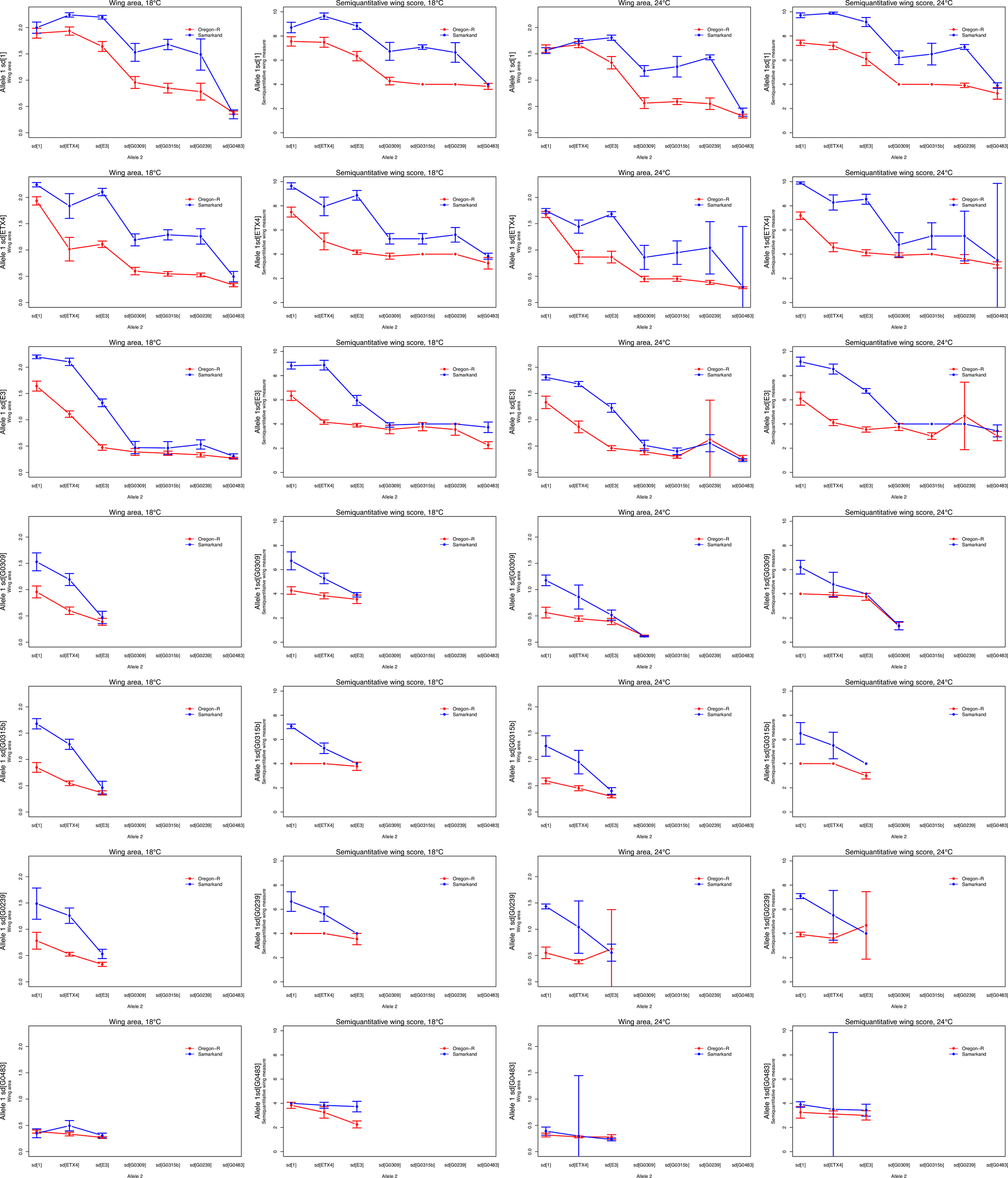

Supplement: S4 Fig — Each panel depicts heterozygous flies carrying allele 1 (labeled on the y-axis) and allele 2 (x-axis). Left two panels: wing area and semi-quantitative wing scores for flies reared at 18°C. Right two panels: flies reared at 24°C. Error bars represent 95% confidence intervals. (TIF) [file pgen.1007075.s005.tif]

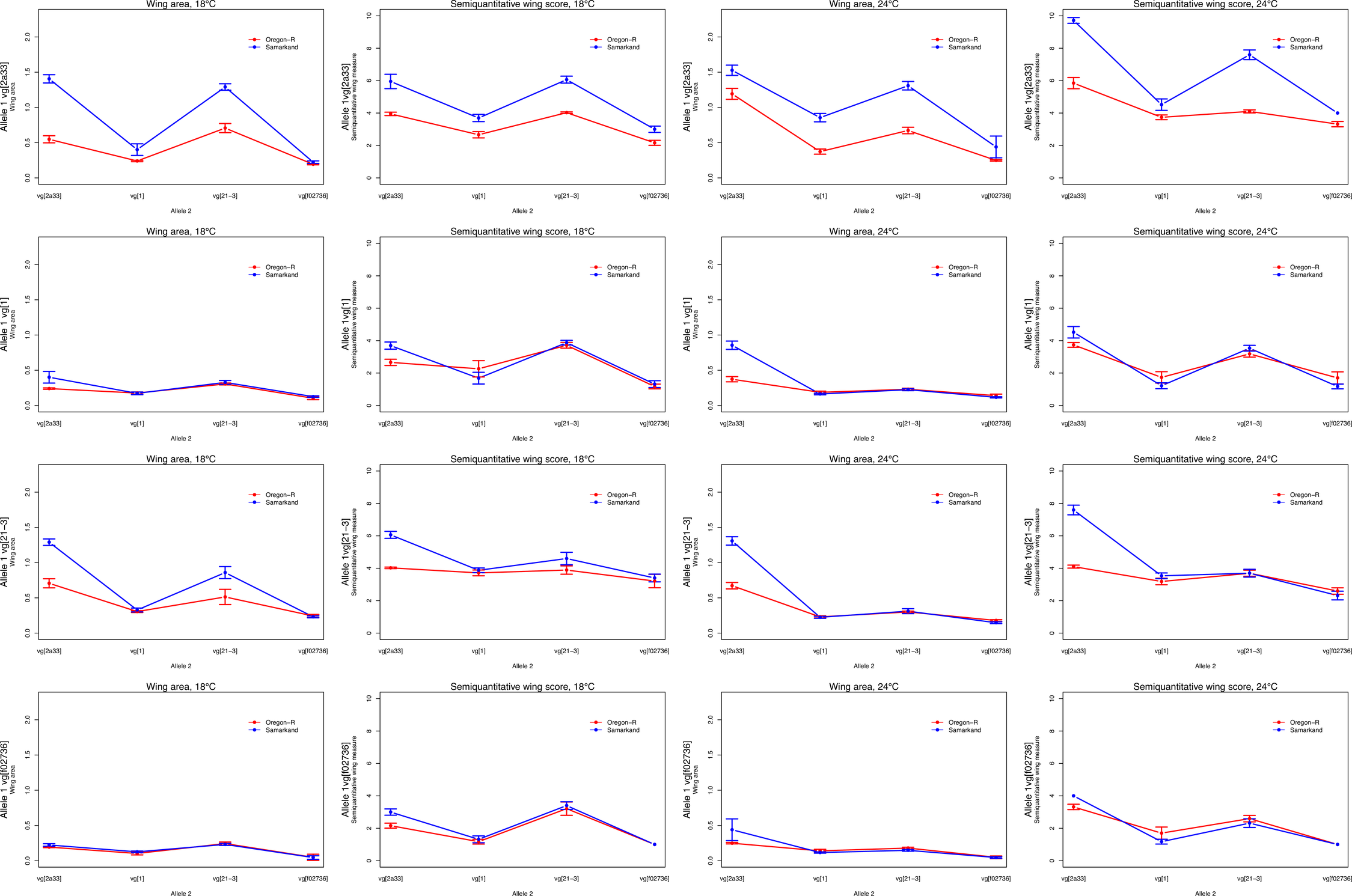

Supplement: S5 Fig — Each panel depicts heterozygous flies carrying allele 1 (labeled on the y-axis) and allele 2 (x-axis). Left two panels: wing area and semi-quantitative wing scores for flies reared at 18°C. Right two panels: flies reared at 24°C. Error bars represent 95% confidence intervals. (TIF) [file pgen.1007075.s006.tif]

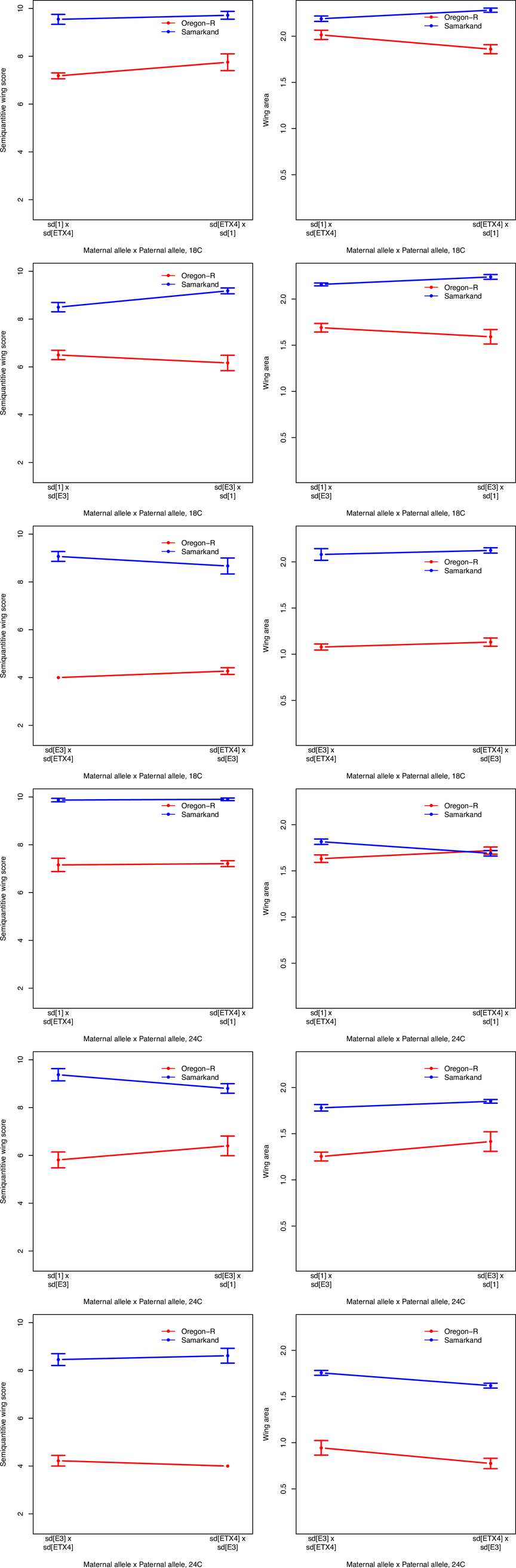

Supplement: S6 Fig — In this case, only female flies were measured, as sd is X-linked and male flies resulting from these crosses are hemizygous for just a single sd allele. Error bars represent 95% confidence intervals. (TIF) [file pgen.1007075.s007.tif]

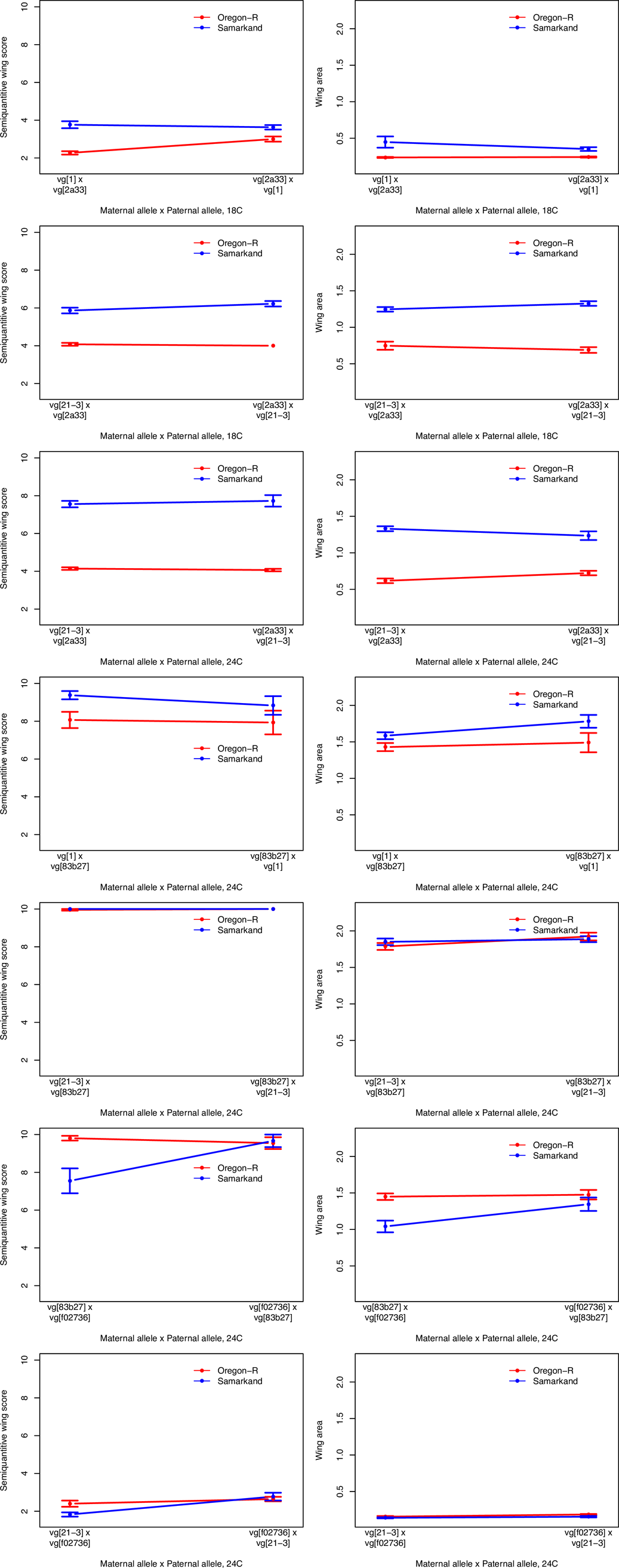

Supplement: S7 Fig — Flies from reciprocal crosses between parents carrying different vg alleles have similar wing phenotypes. Error bars represent 95% confidence intervals. (TIF) [file pgen.1007075.s008.tif]

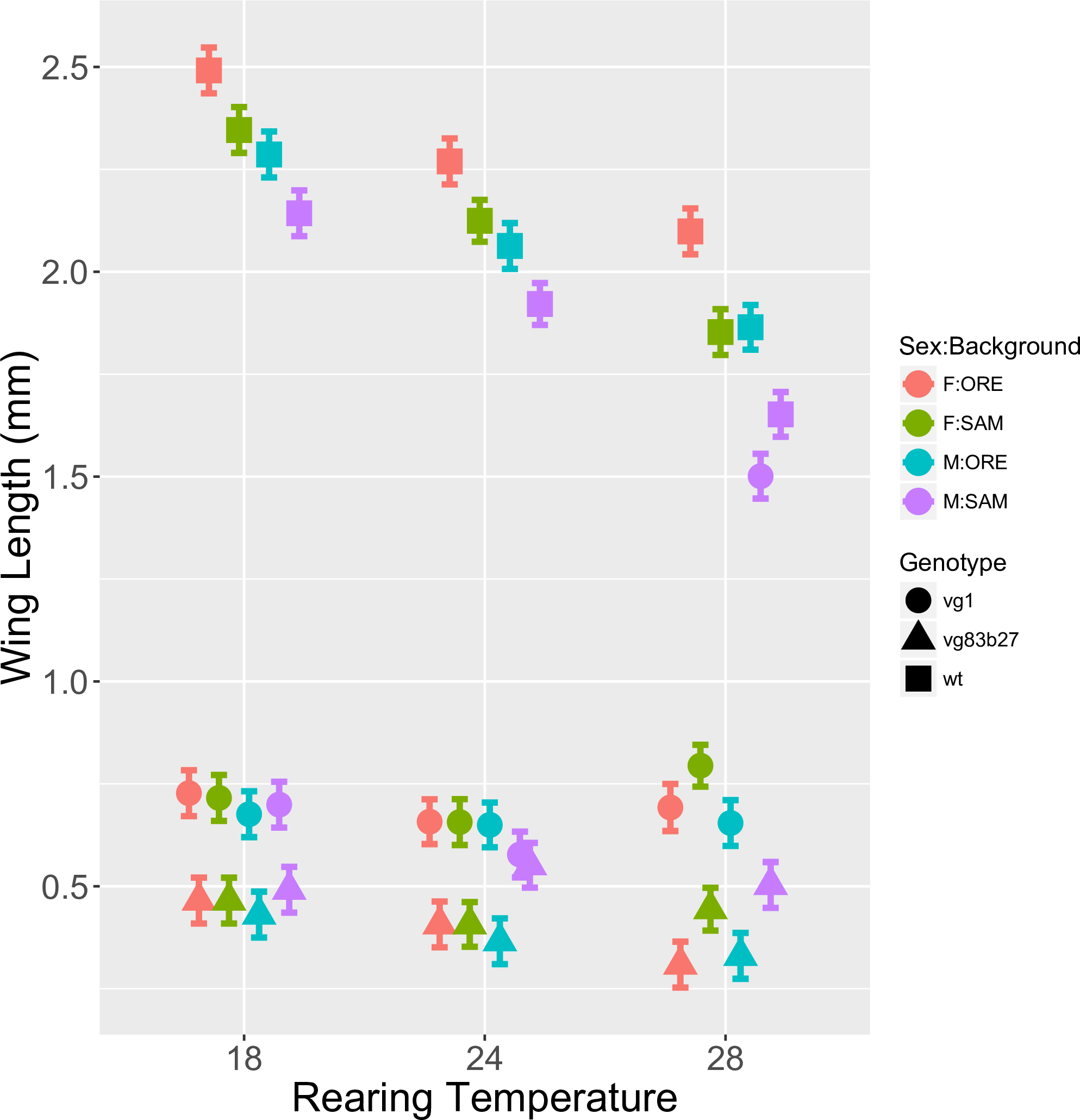

Supplement: S8 Fig — In particular note the phenotype of SAM vg1 males at 28°C (purple circle). Error bars represent 95% CI. (TIF) [file pgen.1007075.s009.tif]

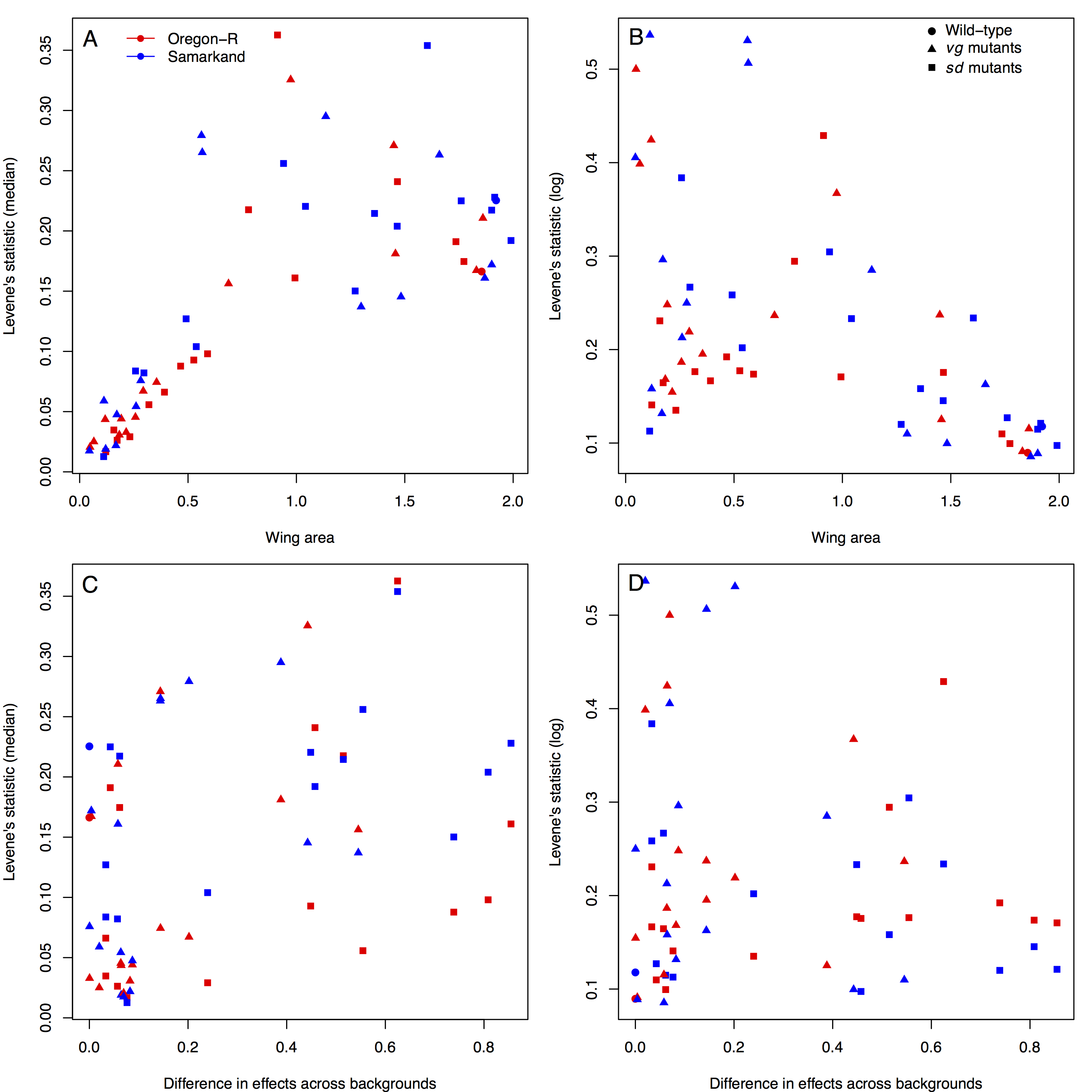

Supplement: S9 Fig — There is a generally curvilinear relationship between the extent of mutational severity (as assessed by reduction in wing area) and intra-genotypic variability (A, B). As can be seen with the log transformed values of Levene’s statistic (B), genotypes with the most severe effects do increase variability, although with considerable among-genotype variation in the within-genotype variability. However, there is only a weak correlation between the background effects and intra-genotypic variability for the median form (C) of Levene’s statistic (Pearson r = 0.41, CI: 0.18,0.6), and very weak for the log transformed measure (D) of Levene’s statistic (Pearson r = -0.09, CI: -0.33,0.16). We also observed little evidence for differences in the two wild type strains (ORE and SAM) in intrinsic variability across all genotypes. We used two forms of Levene’s statistic, one using raw deviations from the genotypic/background medians, and one using log transformed values to assess scaling effects for trait size. (TIFF) [file pgen.1007075.s010.tiff]

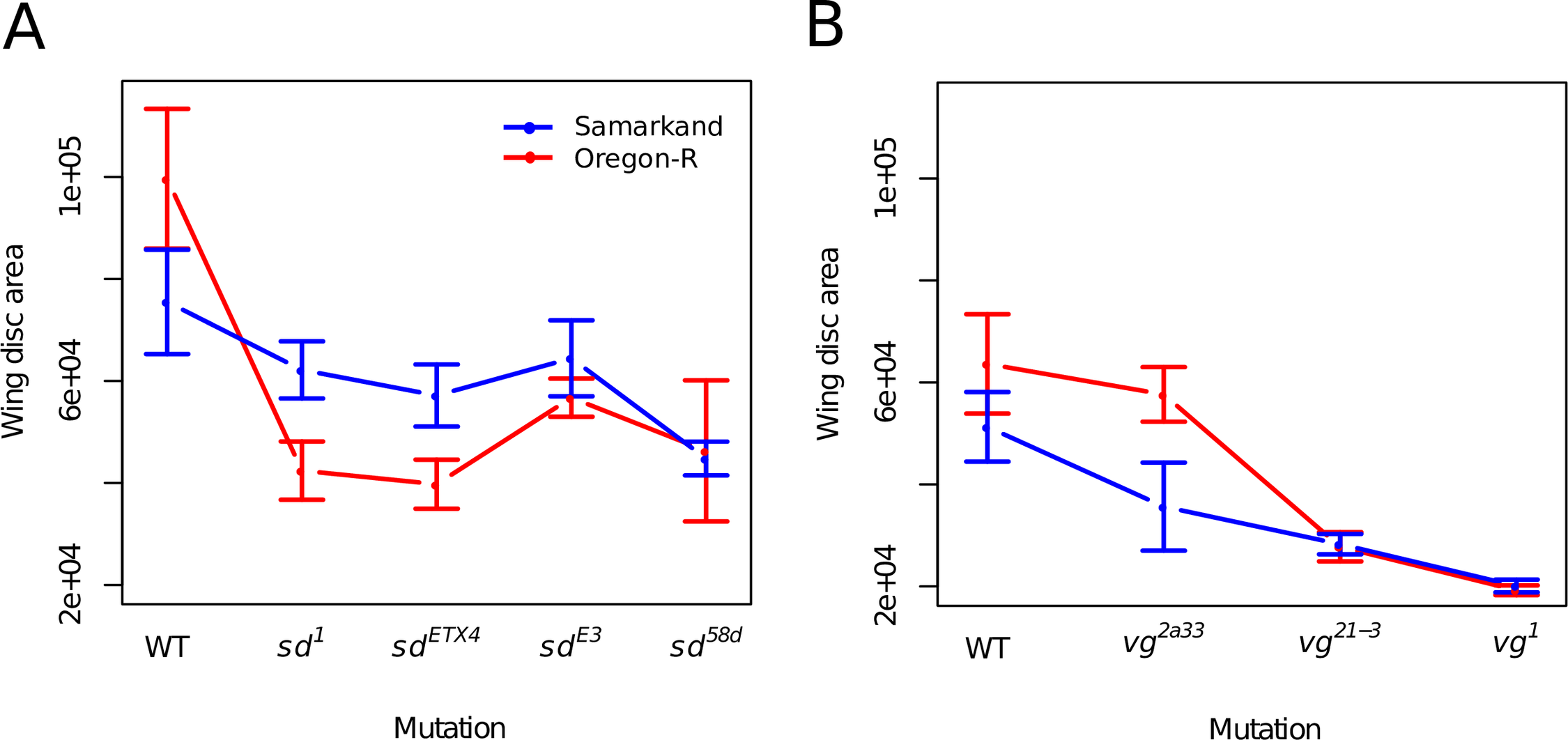

Supplement: S10 Fig — In addition to changes in cell proliferation and spatial distribution of the WG protein (Fig 6), mutant alleles of sd (A) and vg (B) influence pouch size (defined by proximal ring of WG expression as shown in Fig 6A and 6B) in a background dependent manner. Experimental animals were reared separately for the sd and vg allelic series (each with their own sets of control animals), and variation in wing disc size among the wild type discs between A and B likely reflects subtle differences in food batch, and fixation for immunofluorescence. Despite the effects of individual alleles, we do not see a strong association between size of the wing pouch and the final size of the adult wing, compared to patterns of proliferation (Fig 6C and 6E) and spatial distribution of WG protein (Fig 6D and 6F). (TIF) [file pgen.1007075.s011.tif]
